# Love Hug—Functional Validation of Nuptial Pad-Secreted Pheromone in Anurans

**DOI:** 10.3390/ani14111550

**Published:** 2024-05-24

**Authors:** Puyang Zheng, Yuzhou Gong, Bin Wang, Haoqi Yu, Sining Huang, Xun Liao, Jianping Jiang, Jianghong Ran, Feng Xie

**Affiliations:** 1 Chengdu Institute of Biology, Chinese Academy of Sciences, Chengdu 610041, China; zhengpy@cib.ac.cn (P.Z.); wangbin@cib.ac.cn (B.W.); yuhq@cib.ac.cn (H.Y.); huangsn@cib.ac.cn (S.H.); liaoxun@cib.ac.cn (X.L.); jiangjp@cib.ac.cn (J.J.); 2 Key Laboratory of Bio-Resources and Eco-Environment of Ministry of Education, College of Life Sciences, Sichuan University, Chengdu 610065, China; rjhong-01@163.com; 3 University of Chinese Academy of Sciences, Beijing 100049, China; 4 Shanghai Natural History Museum, Branch of Shanghai Science & Technology Museum, Shanghai 200041, China; bobinskygong@163.com; 5 School of Life Science, East China Normal University, Shanghai 200062, China

**Keywords:** anuran, nuptial pad, plethodontid modulating factor, pheromone, behavioral experiment

## Abstract

**Simple Summary:**

This study aimed to investigate the function of the nuptial pad-secreted pheromone in anurans. By conducting transcriptome sequencing of the nuptial pads of the Yunnan pond frog, we obtained transcripts of protein pheromones that were highly expressed in the nuptial pads during the breeding season. These pheromones were homologous to the common frog amplexins and the plethodontid modulating factor (PMF) pheromone of urodeles. By means of recombinant protein expression, we obtained the single subtype of the pheromone and demonstrated its ability to shorten the duration of Yunnan pond frog pairing through behavioral experiments. The results of this study further confirm that urodeles and anurans share the PMF pheromone system. Additionally, this study validates the function of this pheromone in anurans for the first time, confirming that it serves similar functions as in urodeles, shortening the breeding process. Overall, this study provides a paradigm for exploring the function of pheromones in anurans.

**Abstract:**

Chemical communication is an important mode of communication in the courtship and breeding processes of amphibians. In caudates, multiple components of sexual pheromones have been identified and functionally verified. One of these pheromone systems is plethodontid modulating factor (PMF). In anurans, the pheromone called amplexin was found in nuptial pads of ranids and was considered a member of the PMF system, yet its bio-function has not been tested. In this study, we obtained 18 amplexin transcript sequences from nuptial pads of *Nidirana pleuraden* (Amphibia, Ranidae) by transcriptome sequencing and found that the proteins translated by these transcripts are diversified, hydrophilic, and relatively stable. We also acquired a *N. pleuraden* amplexin isoform with the highest expression level in the transcriptome analysis through the prokaryotic expression system. Using two different animal behavioral experimental settings, we have tested the bio-function of the recombinant PMF protein (rPMF) in *N. pleuraden*’s reproduction and found that the rPMF does not attract females but shortens the duration of amplexus significantly. This is the first study to verify the function of the PMF pheromone in Anura, indicating the pervasiveness of chemical communication during breeding in amphibians.

## 1. Introduction

Amplexus is one of the most widespread reproductive behaviors in anurans [1], typically characterized by the male mounting the female’s back, with its forearms pressing under the female’s armpits to hold her tightly, so we figuratively call it “hugging”. This behavior is essential for coordinating egg deposition and sperm release [2,3]. During the breeding season, many male frogs or toads develop keratinized spines on their nuptial pads on the thumbs or forearms, like in *Rhinella marina* [4], *Ptychohyla hypomykter* [5] and *Rana temporaria* [6]. It is widely believed that nuptial pads strengthen the male’s grip on the female during amplexus [3]. Histomorphological studies have also shown that multicellular, alveolar glands with granular secretive products are presented beneath the surface of the nuptial pads, sharing characteristics with the well-known pheromone glands in salamanders [7]. This suggests that nuptial pads may synthesize sexual pheromones during the breeding season. Instead of via vomeronasal organ, pheromones produced in the mental gland of some plethodontid salamanders are transmitted directly into the female’s blood circulation system by dermal attrition [8]. Interestingly, examining post-amplexus female frogs reveals that their ventral skin is often abraded at the site where the male’s spiny nuptial pads contact [6], which suggest that a similar pheromone transmission pattern might exist in anurans.

Chemical communication has been proven widespread in the courtship and reproduction processes of amphibians [9,10]. In caudates, multiple courtship pheromones have been identified and functionally validated [9,10,11,12,13], most of which are proteins or peptides [14]. Early studies suggested that in anurans, vocal communication was the primary means of interaction [2,3,15]. However, increasing evidence indicates that chemical communication also plays a significant role in the reproduction and courtship behaviors of frogs and toads [5,16,17,18,19].

Currently, three major pheromone systems exist in amphibians: plethodontid receptivity factor (PRF) [9], plethodontid modulating factor (PMF) [20] and soderfin precursor-like factor (SPF) [21]. Among them, PMF was first found in the mental gland of male plethodontid salamanders [20], its members weigh 7 to 10 kDa, and it structurally belongs to the three-finger protein (TFP) superfamily. Animal behavioral experiments indicate that the PMF pheromone enhances female receptivity and reduces the duration of female courtship behaviors in plethodontid salamanders [20]. By 2013, Willaert et al. identified a set of pheromones in the nuptial pads of *R. temporaria* (Amphibia, Anura, Randidae), which they named amplexin(s). These secretive proteins showed homology with PMFs and were speculated to play a role in reducing amplexus duration. However, the biofunction of amplexins requires further verification [6].

To gain a deeper understanding of the biological function of pheromones in anurans, our study focuses on investigating the potential similarity between the secretions of the most prevalent breeding gland, the nuptial pad, and PMFs in caudates, as hypothesized by Willaert [6]. We selected another ranid species, the Yunnan pond frog (*Nidirana pleuraden*), which develops pronounced nuptial pads during the breeding season, as our target. We first performed a comparative transcriptome analysis of its nuptial pads and dorsal skin to obtain the tissue-specific transcript sequences. Subsequently, we screened out the transcripts that show sequence similarity to the reported PMF pheromones. Then, we selected the transcripts with the highest expression level for prokaryotic expression to obtain the recombinant PMF protein. Finally, we designed two sets of behavioral experiments to test the function of the recombinant pheromone.

## 2. Materials and Methods

### 2.1. Animals

In June 2017, three adult male *N. pleuraden* were collected from the Caohai National Nature Reserve in Bijie, Guizhou, China. The frogs were anesthetized using MS22 solution, and then their nuptial pads and dorsal skin were swiftly excised on ice. The excised tissues were immediately placed into RNase-free centrifuge tubes, flash-frozen in liquid nitrogen and subsequently stored at −80 °C for transcriptome sequencing.

For behavioral testing, 30 females *N. pleuraden* were captured in the reserve during the nights of 21 to 25 August 2022 and 22 to 26 July 2023. Two sets of ethological experiments were conducted separately in different years. On the day of the experiment, animals were collected individually and kept in covered plastic containers (15 × 10 × 6 cm^3^) with damp paper towels inside. After completing a set of experiments on the following night, the animals were released back into the wild. Given this sampling–experiment–release procedure, the possibility that the same individual was introduced into multiple sets of experiments cannot be excluded.

The frogs used in this study were all sexually mature. Male frogs were located by their advertisement calls, and female frogs were identified by having a snout-vent length greater than 55 mm and exhibiting abdominal swelling (indicative of gravidity). All experimental procedures involving animals were conducted in accordance with the regulations established by the Animal Care and Use Committee of the Chengdu Institute of Biology, Chinese Academy of Sciences (CIBDWLL2021014).

### 2.2. Transcriptome Sequencing and Analysis

Nuptial pads and dorsal skin samples from male *N. pleuraden* specimens were collected for transcriptome sequencing during breeding season. Each type of tissue included three replicates for a total of six samples. Total RNA was isolated using the TRIzol^®^ reagent (Invitrogen, Carlsbad, CA, USA) followed by treatment with RNase-free DNase I (Promega, Madison, WI, USA) according to the manufacturers’ protocols. The concentration of extracted RNA was detected with Nanodrop2000 (Agilent, Santa Clara, CA, USA), and the integrity was examined with Agient2100, LabChip GX (Perkin Elmer, Hopkinton, MA, USA). Illumina RNA-seq libraries were prepared for 6 samples and sequenced on Illumina NovaSeq 6000 platform (Illumina, San Diego, CA, USA) with a PE150 strategy following the manufacturer’s instructions.

The expression level of predicted transcripts in each RNA-seq library was calculated as the fragments per kilobase of exon model per million mapped fragments (FPKMs) using the following formula: FPKM = (exon mapped fragments × 10^9^)/(total mapped fragments × exon length). To identify the differentially expressed genes (DEGs), we compared FPKM values from nuptial pads and dorsal skins. DEGs were identified as |log2(fold change)| > 1 and BH corrected *p* < 0.01 by the DESeq2 package v1.38.3 [22]. The Trinotate v3.2.1 annotation tool [23] was used to search seven major databases for functional annotation, the software used in the retrieval and the set threshold (*e*-value); see Supplementary Table S1.

GO enrichment was performed on the genes highly expressed in the nuptial pads relative to the dorsal skin and elimination of redundancy by ReviGo v1.8.1 [24].

### 2.3. Protein Sequence Alignment and Amino Acid Characteristic Analysis

After annotation of the nuptial pads transcripts, we selected the sequences annotated as amplexin or PMF and screened the domains of these sequences using InterPro [25]. Sequences with high expression and complete domains in the nuptial pads were used for subsequent analysis. Multiple sequence alignment of amino acid sequences was conducted using the MAFFT software (version 7), utilizing the L-INS-I model for the alignment [26]. The *R. temporaria* amplexins (GenBank accession nos KC282376–KC282380) and two caudate PMFs (GenBank accession numbers AEO22663 and ABI48851) were used for multiple sequence alignment. Visualization of the alignment results was performed using GeneDoc v2.7 [27]. Amino acid sequence identity was calculated in the software BioEdit v7.0.9 [28] using the aligned sequences. The theoretical molecular weight, isoelectric point, instability index, and average hydrophobicity, among other characteristic parameters of the proteins, were calculated by submitting the sequence information to the ExPASy website and using the ProtParam tool (https://web.expasy.org/protparam/ (accessed on 6 April 2024)).

### 2.4. Recombinant Expression of Pheromone Protein

Recombinant PMF (rPMF) was expressed as a precursor protein with a cleavable N-terminal 6xHis affinity tag. We chose the Npamplexin05 (GenBank accession number PP583595) for expression because this isoform is the most abundantly expressed subtype in the nuptial pad of males. The bridge polymerase chain reaction technique (Overlap PCR) was used to fuse the Trx sequence, His purification tag, and enterokinase sequence at the N-terminus of the Npamplexin05. The fusion fragment was inserted into the pET32a (+) vector after double digestion with KpnI and HindIII, and the recombinant plasmid was transformed into Rosetta (DE3) cells. Single-clone colonies were inoculated into 100 mL LB (Amp: 100 μg/mL) and activated and expanded at 37 °C, 185 rpm until the culture OD600 reached about 0.6. Then, 0.1 mM IPTG was added to induce expression at 16 °C, 185 rpm for 8 h. The cells were collected by centrifugation and lysed, and the supernatant was purified using Ni-NTA affinity chromatography to obtain the Trx-His-EK-PMF fusion protein. The protein was quantified, and an appropriate amount was incubated with enterokinase at 20 °C for 16 h to cleave off the N-terminal fusion fragment. The protease digestion system was then subjected to Ni-NTA affinity chromatography to recover the target protein PMF. Purified rPMFs were taken for SDS-PAGE analysis.

### 2.5. Behavioral Experiments and Data Analysis

Two sets of behavioral tests were conducted, each consisting of 30 trials. A network surveillance camera equipped with an infrared module was installed above the test apparatus and operated through IPClient V1.0.7.57 software to record animal behavior. The following is a description of the two sets of tests.

The first group of tests was conducted to evaluate whether the rPMF protein is attractive to female *N. pleuraden*. The experimental aquarium was made in a do-it-yourself manner: a PVC rainwater guiding trough with a length of 1 m and a rectangular cross-section (18 cm wide) was sealed at both ends with matching plastic covers. Black markers were used to draw lines along the edges of the trough, dividing it into three areas: the areas extending 10 cm from the central line of the trough to both sides were designated as “waiting areas”, and the remaining areas on the left and right sides of the trough (40 × 18 cm^2^) were designated as ‘choice areas’ (Supplementary Figure S1). If the test individual spends a significantly longer total time in one choice area than in the other, it indicates a preference for the stimulus placed in that choice area. Before the test began, tap water that had been left to stand overnight was added to the experimental trough until the water depth reached 6 cm. Then, the test female frog was placed at the center of the waiting area and covered with a metal wire mesh box to restrict its movement. At the same time, 10 μL (ca. 5 μg) rPMF was injected into a sponge block (1 cm^2^), and then the sponge block was attached to one end of the experimental tank (below water level); at the other end is a sponge block injected with an equal volume of water (to avoid position effects, the end of the trough where the stimuli were placed was randomly determined in each trial), and the camera’s recording function was turned on. After a 10-min animal adaptation period, the metal wire mesh box was removed, allowing the test female to move freely in the experimental trough. Once the female left the waiting area, a 10-min timer started. After the timing ended, the test female and the stimuli were removed from the trough, the camera’s recording function was turned off, and the trough was carefully cleaned with a detergent. After cleaning, the trough was rinsed several times with standing tap water before proceeding to the next trial.

Another group of tests was conducted to evaluate the effect of the rPMF protein during the amplexus process. The experimental tank was a white PVC basin (50 × 30 × 25 cm^3^). Before the experiment began, tap water that had been left to stand overnight was added to the tank until the water depth reached 6 cm. Subsequently, a 1 mL syringe needle was used to gently puncture the skin under the female’s armpit, and after injecting 10 μL (ca 5 μg) rPMF protein solution, the female was quickly embraced with a model frog and then placed in the tank with the camera’s recording function turned on (Supplementary Figure S2). After the female showed struggling movements, the time was recorded and the camera was turned off. The test female and model frog were then removed from the tank, and the tank and model frog were carefully cleaned with a detergent. After cleaning, they were rinsed several times with standing tap water. The same process was repeated for another female with the difference being that the PMF protein solution was replaced with distilled water, treating the two frogs as one experiment.

Based on the activity rhythm of the *N. pleuraden*, the experiments were conducted from 20:00 to 02:00 each day. Behavioral experiments were conducted in the yard of a farmhouse near the Caohai Nature Reserve (26.873391° N, 104.225566° E, 2178.84 m asl), where environmental factors are similar to the natural breeding habitats of the animals. The first set of experiments commenced on 23 August, 24 August, and 25 August 2022. The second set of experiments was conducted on 24 July, 25 July, and 26 July 2023. The dosage of rPMF in all experiments was determined based on the research conducted by Houck et al. on recombinant PRF [29].

We used the player plugin built into IPClient to watch the animal behavior recorded by the camera. For the choice experiment, we started timing when the test female left the waiting area and tallied the total time the animal spent on both stimulus sides of the experimental tank (crossing the midline of the tank is used as an indicator for changing stimulus sides). For the amplexus experiment, we started timing immediately after the test female was placed in the tank and stopped timing when the female showed clear struggling movements and recorded the intermediate time.

We compared two sets of data obtained from experiments with the same design (corresponding to the time the test animals were active on different stimulus sides, n = 30). Since the data did not conform to normality, a Wilcoxon rank-sum test was used. All tests were two-tailed with a significance level (*p*) of 0.05. Statistical comparisons of the data were performed with R, and the bar graphs of the behavior experiment results were drawn using the ggplot2 package [30] in R.

## 3. Results

### 3.1. PMF Sequence Acquisition

**Figure 1:**
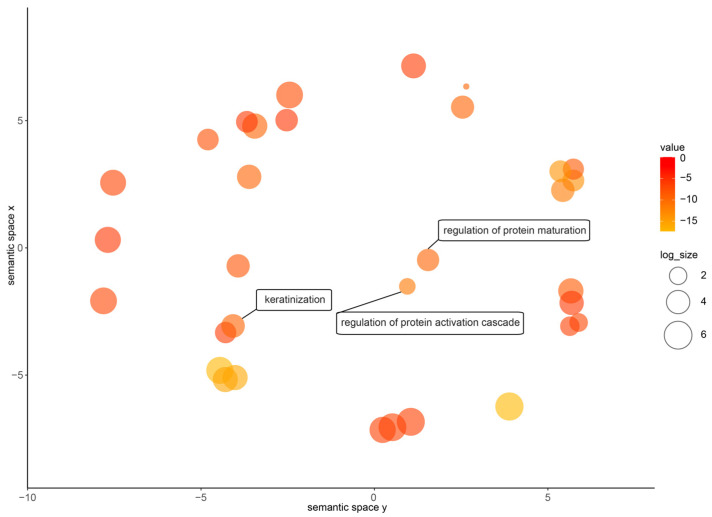
Enriched GO terms of upregulated genes in nuptial pad of *N. pleuraden* with interested term highlighted.

Quantifying gene expression helps to describe the degree of difference between different sequencing samples. After normalizing the reads count by calculating the probability of hypothesis testing and correcting for multiple testing, we found that there are more upregulated genes (810) in the nuptial pad of *N. pleuraden* compared to its dorsal skin (Supplementary Table S2). The GO enrichment results of these genes (Figure 1) showed that they were enriched in protein-related GO terms such as regulation of protein maturation (G0:1903317) and regulation of protein activation cascade (GO:2000257), indicating that the nuptial pad of *N. pleuraden* had more vigorous protein synthesis activity than the dorsal skin. At the same time, they were also enriched in regard to keratinization (G0:0031424), which was consistent with the nuptial spines growing on the surface of the nuptial pad in the breeding season. Meanwhile, by searching the annotation information of these highly expressed genes (Supplementary Table S3) and conducting domain screening, we found 18 sequences annotated as amplexin in the nuptial pads of the *N. pleuraden*. The most highly expressed gene (Npampliexin05) ranks within the top 10% of upregulated genes in terms of expression levels. The sequence accession numbers are shown in Table 1.

### 3.2. Amino acid Sequence Characteristics of N. pleuraden PMF

**Figure 2:**
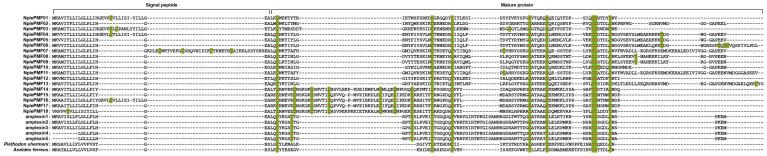
The multiple sequences alignment result of *N. pleuraden* PMFs. The green background highlights the cysteine among aligned sequences.

Multiple sequence alignment results (Figure 2) show that the PMF pheromone sequences of the *N. pleuraden* can generally be divided into a signal peptide segment of 19 to 60 amino acids and a subsequent mature protein segment of 71 to 108 amino acids, although some sequences show insertions. Comparing with the *R. temporaria* amplexin, the cysteine distribution of *N. pleuraden* PMF was consistent with that of the caudate PMF.

The amino acid identity (Table 2) of *N. pleuraden* PMF was relatively low, about 34.9%, while that of *R. temporaria* was relatively high, about 86.8%, which is mainly due to the single amino acid insertions occurring in the middle of the *N. pleuraden* PMF sequences and large sequence-specific regions at the signal peptide region and tail.

After removing the signal peptide segment, the molecular weight of *N. pleuraden* PMF pheromones ranges between 8.14 and 12.36 kDa. The isoelectric point varies widely from 4 to 8, indicating that these pheromones have subtypes that are negatively charged, uncharged, or weakly positively charged in neutral solutions. The instability index also varies significantly, ranging from 20 to 45, suggesting that some subtypes are stable while others are not. The total average hydrophilicity values are all negative, indicating that the *N. pleuraden* amplexins are water-soluble. Additionally, despite significant variations in amino acid sequences, the estimated half-life of these pheromones is overwhelmingly 5.5 h (Table 3), suggesting that the *N. pleuraden* PMFs may have long residual action.

### 3.3. Recombinant Expression of N. pleuraden Amplexin

**Figure 3:**
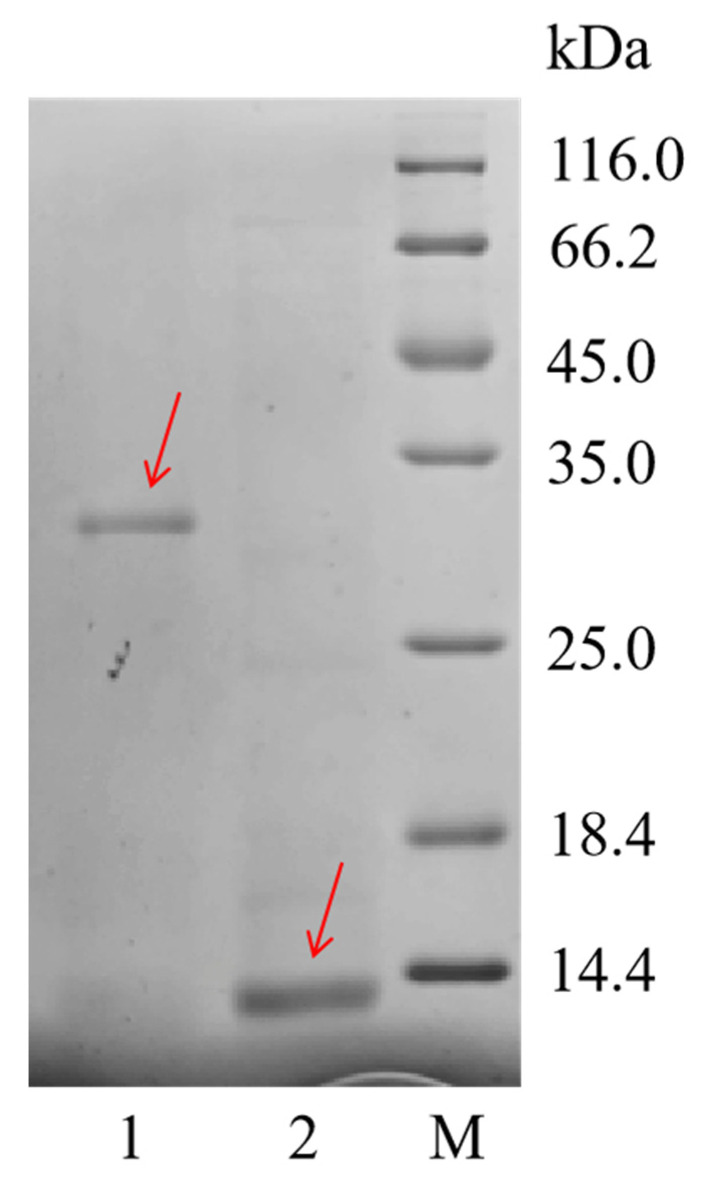
SDS-PAGE image of rPMF protein. Lane M: protein molecular weight standard; Lane 1: Purified protein before digestion; Lane 2: Purified protein after digestion. Red arrows indicate bands of recombinant proteins.

According to the differential expression results of the transcriptome, we selected the sequence with the highest expression level among all PMF-related genes, Npamplexin05 (average FPKM = 6093), for PMF protein recombinant expression. SDS-PAGE results (Figure 3) showed that the undigested purified protein molecular weight was about 31 kDa, and after digestion, the purified protein molecular weight had obvious bands at about 11 kDa, which was consistent with the prediction, indicating that the recombinant protein expression experiment was successful. Finally, we harvested 1 mg of amplexin protein, which is about 11 kDa in size and 90% in purity.

### 3.4. Functional Verification of N. pleuraden Amplexin

**Figure 4:**
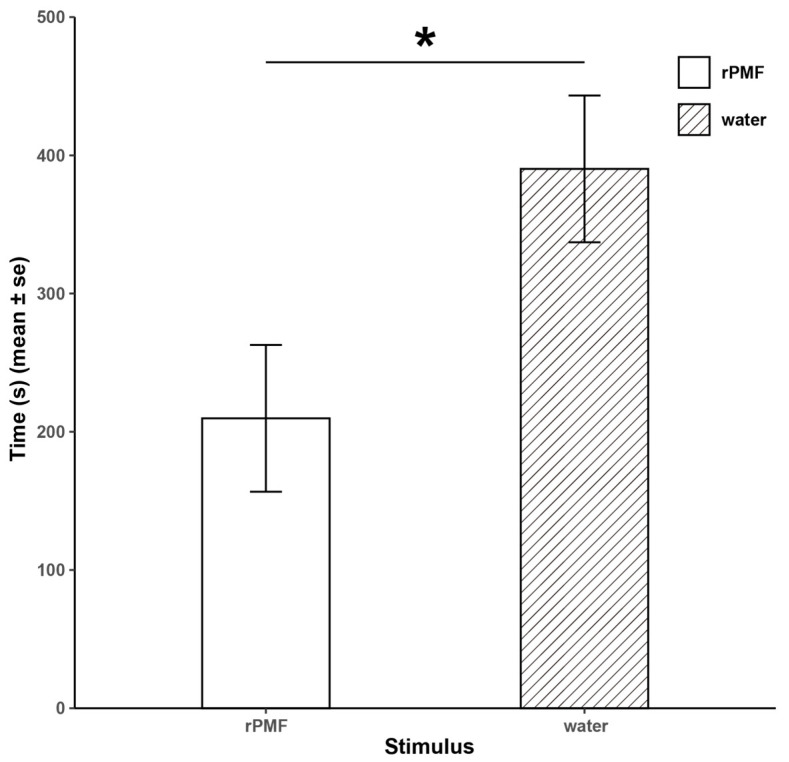
Results of the mate choice preference experiment. * *p* < 0.05.

In the mate choice preference experiment (Figure 4), we observed that female *N. pleuraden* spent a significantly longer time on the side with distilled water than on the side with the rPMF (390.2 ± 53.09 s vs. 209.7 ± 53.09 s, Z = 2.91, *p* < 0.05). Additionally, 22 test frogs immediately swam to the end with distilled water after adapting to the environment, suggesting that PMF has a repelling effect on females.

**Figure 5:**
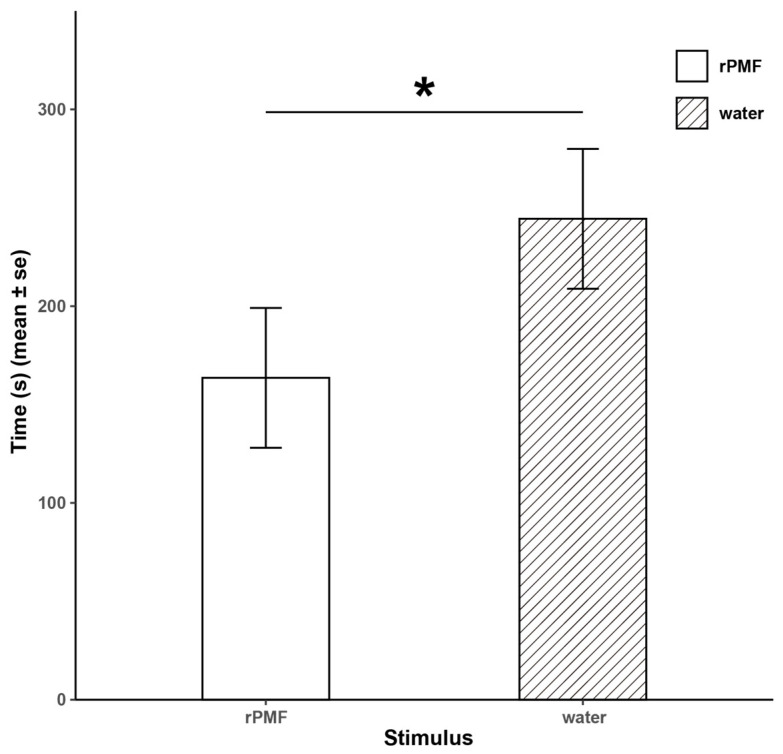
Results of the amplexus experiment. * *p* < 0.05.

In the amplexus experiment (Figure 5), the time it took for the female frogs injected with PMF to start struggling was significantly shorter than for those injected with distilled water (163.6 ± 35.53 s vs. 244.4 ± 35.53 s, Z = 2.61, *p* < 0.05). The results of the two experiments corroborate each other, indicating that PMF plays a role in shortening the amplexus process in *N. pleuraden*.

## 4. Discussion

The nuptial pad is the most thoroughly studied amphibian reproductive gland to date, and it is reported in both Anura and Caudata. During the breeding season, the conical protrusions of the nuptial pad’s dermis penetrate the epidermal germinative layer to form breeding spines [8,31,32]. Histological studies indicate that the nuptial pad shares a similar composition with the pheromone-producing gland in Caudata [7]. The discovery of amplexin in the nuptial pads of the *R. temporaria* suggests that the secretion of the nuptial pad is proteinaceous pheromones, which are homologous to the PMF in Caudata [6]. By comparing the transcriptomes of the nuptial pad and the dorsal skin of *N. pleuraden* during the breeding season, we found 18 genes highly expressed in the nuptial pad annotated as amplexin, consistent with findings in the *R. temporaria*, indicating the similarity of secretions in the nuptial pads across anurans. The results of multiple sequence alignment showed that both *N. pleuraden* and *R. temporaria* amplexins had homology and similar cysteine skeleton with PMFs in tailed amphibians. Based on sequence homology and the cysteine skeleton, we believe that amplexin should be considered as a member of the PMF pheromone system.

The PMF pheromones are reported to have multiple isoforms in Caudata, and the discovery of amplexins in *R. temporaria* suggests that multiple subtypes of PMF also exist in anurans. We have found 18 amplexins in the nuptial pad of the *N. pleuraden*, further confirming the diversification of the PMF system. Interestingly, although multiple subtypes of PMFs are present in different amphibian groups, the identity of these PMF sequences varies significantly between groups. For instance, in studies on the red-legged salamanders, *Plethodon shermani*, the PMFs show about 30% amino acid sequence identity [33]. We calculated the amino acid sequence identity in auran’s PMFs and found approximately 40.2% in *N. pleuraden* and 86.8% in *R. temporaria*, suggesting a high degree of diversity in PMFs among amphibians [34]. This diversity is probably the result of long-term co-evolution with multiple female receptors. Diversity in sequence and structure may help male pheromones bind to more potential female receptors or respond quickly to slight changes in the receptor sequence [35]. It is worth noting that the sequence identity of *R. temporaria* amplexins is much higher than that of the other amphibian species’ PMFs. The discovery process of *R. temporaria* amplexins was through the construction of a cDNA library of the nuptial pad and the use of primers for RACE PCR. In this process, only one individual of *R. temporaria* and one primer designed according to the signal peptide were used [6]. We speculate that other PMF subtypes may be ignored in this process. With the development of omics technology, the use of multi-omics in the study of pheromones in the future will be conducive to achieving efficient pheromone identification. From a physicochemical perspective, the isoelectric point and instability index of the *N. pleuraden* amplexins show significant variation, reflecting its lower sequence similarity. Moreover, PMF belongs to the three-finger protein superfamily, most of which are characterized by the sequence variability and structural stability maintained by disulfide bonds [36]. Multiple sequence alignments indicate that the *N. pleuraden* and the *R. temporaria* amplexins as well as the caudates’ PMF all have a stable cysteine skeleton, suggesting a potentially similar function.

PMF has been reported in all species of Plethodontidae [33,34,37,38], where its various subtypes in combination were sufficient to increase female sexual receptivity, achieving the effect of shortening the female’s tail-straddling walk time [20]. It was previously thought to have originated 42–50 mya after the divergence of the species of the family Plethodontidae and Salamandridae [39,40], but the homologous amplexins was found in the *R. temporaria* [6] and in the *N. pleuraden*, suggesting that the origin of this pheromone system should be dated back to 297–300 mya before the separate evolution of the ancestors of the caudates and anurans [41,42].

The results of a previous study show that a single subtype does not alter female behavior; a mixture of PMFs lacking the three main subtypes (PMF-G, PMF-H, PMF-I) can actually decrease female sexual receptivity and prolong courtship duration [43,44]. This not only suggests that the function of this class of pheromones may not be similar to SPF system’s gender attraction [9,45] but also indicates that the functions of the subtypes within this pheromone system are non-redundant and act in a synergistic manner, which may have promoted the increasingly complex and diverse chemical signaling in male plethodontid salamanders. Willaert et al. discovered amplexin in the nuptial pads of *R. temporaria* and based on its homology to PMF sequences in caudates [6] speculated that it could have a similar function; the amplexin enters the female’s circulatory system directly through abrasions caused by nuptial pad friction, thereby reducing amplexus duration and decreasing the risk of predation during mating. Our experimental results imply that PMF functions not only via the circulatory system but also through the animals’ olfactory system. The highly expressed PMF subtype in the nuptial pads of *N. pleuraden* does not exhibit an attractive effect on females in the mate choice experiment, similar to findings in caudates, confirming that PMF does not function as a gender attractant like SPF. Moreover, in subsequent amplexus experiments, we found that females injected with rPMF typically escaped faster than those injected with distilled water, which is consistent with the hypothesis suggested by Willaert et al. about amplexin’s function [6]. Therefore, we believe that the function of PMF in *N. pleuraden* is to reduce the duration of amplexus. Functionally, whether in caudates or anurans, the role of PMF pheromones is essentially consistent, indicating that this pheromone system has widespread applicability. The observation in caudates that some single subtypes do not function [43,44] did not occur in our study. We speculate the primary reason is that we chose the most highly expressed sequence in the nuptial pads for recombinant protein expression, which is the main subtype of *N. pleuraden* amplexin, thus exhibiting functionality in subsequent behavioral experiments. However, the function of interaction of multiple PMF subtypes in *N. pleuraden* remains to be further verified.

## 5. Conclusions

In conclusion, our study further proves that both caudates and anurans share the PMF pheromone system, and both are protein pheromones with the same cysteine skeleton structure. At the same time, the function of PMF pheromones in anurans was verified for the first time, and it was confirmed that these pheromones had a similar function in both caudates and anurans, shortening the reproductive process. In the Caudata, male salamanders deliver PMF pheromones through ‘biting’ by presenting their submandibular (mental) gland to females, while in the Anura, male frogs deliver pheromones through ‘hugging’ by pressing their nuptial pad against females.

## Figures and Tables

**Table 1:** ID, name and expression information of *N. pleuraden* PMF.

Transcript ID	Translated Protein Name	Expression Level (Average FPKM)	Accession Number
Np_f2p60_635_transcript36823_g2	Npamplexin01	18.0	PP583591
Np_f6p60_691_transcript36243_g1	Npamplexin02	110.0	PP583592
Np_f5p60_577_transcript37403_g1	Npamplexin03	16.0	PP583593
Np_f2p60_624_transcript36920_g2	Npamplexin04	50.3	PP583594
Np_f7p60_783_transcript35402_g2	Npamplexin05	6093.0	PP583595
Np_f3p60_601_transcript37151_g1	Npamplexin06	79.7	PP583596
Np_f2p33_731_transcript35890_g1	Npamplexin07	33.7	PP583597
Np_f2p15_911_transcript34472_g1	Npamplexin08	410.7	PP623143
Np_f2p53_997_transcript33771_g1	Npamplexin09	126.3	PP623144
Np_f2p60_533_transcript37873_g1	Npamplexin10	583.3	PP623145
Np_f4p60_809_transcript35147_g1	Npamplexin11	620.3	PP623146
Np_f7p60_745_transcript35648_g1	Npamplexin12	620.3	PP623147
Np_f9p60_755_transcript35756_g1	Npamplexin13	1921.3	PP623148
Np_f11p60_818_transcript35677_g1	Npamplexin14	2115.0	PP623149
Np_f2p16_866_transcript34815_g1	Npamplexin15	1658.0	PP623150
Np_f2p60_865_transcript34782_g1	Npamplexin16	45.7	PP623151
Np_f3p60_896_transcript34533_g2	Npamplexin17	1996.0	PP623152
Np_f4p60_854_transcript34833_g1	Npamplexin18	9.0	PP623153

**Table 2:** Amino acid sequence identity of amphibian PMFs (%).

Sequences	Npamplexin02	Npamplexin05	Npamplexin08	Npamplexin14	Npamplexin15	Npamplexin17	Npamplexin18	amplexin1	amplexin2	amplexin3	amplexin4	amplexin5	*P. shermani*	*A. ferreus*
Npamplexin02														
Npamplexin05	47.8													
Npamplexin08	43.4	47.4												
Npamplexin14	22.7	22.5	21.1											
Npamplexin15	20.8	21.3	22.0	62.9										
Npamplexin17	20.8	21.3	22.0	61.2	98.3									
Npamplexin18	17.2	13.9	15.2	46.4	42.4	42.4								
amplexin1	28.3	30.1	31.2	26.4	23.7	24.4	19.2							
amplexin2	27.5	30.1	30.4	26.4	23.7	24.4	18.5	90.3						
amplexin3	28.3	30.9	31.2	27.1	24.4	25.1	19.2	87.5	97.1					
amplexin4	23.3	25.3	26.4	21.4	19.4	20.1	17.8	83.6	83.6	81.7				
amplexin5	23.3	26.1	26.4	20.7	18.7	19.4	17.8	78.8	86.5	84.6	94.7			
Plethodon shermani	16.8	19.3	20.1	18.1	15.8	15.8	14.1	20.3	21.2	20.3	17.5	18.5		
Aneides ferreus	17.6	17.6	17.6	18.1	16.6	15.8	15.7	22.2	23.1	23.1	20.3	20.3	38.8	

**Table 3:** Characteristic of the mature pheromone proteins in *N. pleuraden*.

Transcript ID	Number of Amino Acids	Molecular Weight	Theoretical pI	Estimated Half-Life (Mammalian Reticulocytes, In Vitro)	Instability Index	Grand Average of Hydropathicity
Npamplexin01	71	8478.61	7.66	5.5 h	24.74	−0.682
Npamplexin02	90	10,377.82	8.15	5.5 h	45.27	−0.891
Npamplexin03	70	8139.16	5.73	5.5 h	19.74	−1.01
Npamplexin04	96	11,008.44	4.46	5.5 h	38.8	−0.365
Npamplexin05	96	11,008.44	4.46	5.5 h	38.8	−0.365
Npamplexin06	108	12,360.1	4.38	5.5 h	43.35	−0.121
Npamplexin07	106	11,824.42	4.98	5.5 h	42.47	−0.347
Npamplexin08	96	11,016.67	7.65	5.5 h	42.85	−0.489
Npamplexin09	96	1097.55	6.74	5.5 h	44.56	−0.531
Npamplexin10	82	9268.46	4.59	5.5 h	47.73	−0.722
Npamplexin11	117	12,912.49	4.56	5.5 h	52.50	−0.393
Npamplexin12	72	8451.72	8.15	5.5 h	39.52	−0.578
Npamplexin13	110	12,299.89	4.74	0.8 h	63.05	−0.610
Npamplexin14	105	11,926.81	8.21	5.5 h	52,26	−0.549
Npamplexin15	104	11,887.83	8.56	1 h	54.75	−0.560
Npamplexin16	104	11,887.83	8.56	1 h	54.75	−0.560
Npamplexin17	104	11,873.80	8.56	1 h	57.01	−0.561
Npamplexin18	104	12,036.82	8.40	5.5 h	64.80	−0.930

## Data Availability

All the raw sequencing data generated in this study are available in the National Center for Biotechnology Information database under the BioProject number PRJNA1098347. The data are publicly available as of the date of publication.

## Supplementary Materials

The following supporting information can be downloaded at: https://www.mdpi.com/article/10.3390/ani14111550/s1, Figure S1: Diagram of the setup of animal preference trials; Figure S2: (a) A model frog for amplexus experiments; (b) an experiment in progress; Table S1: The databases and software used for non-redundant transcripts function annotation; Table S2: Upregulated genes in the nuptial pads of *N. pleuraden* and their annotation information; Table S3: GO enrichment of upregulated genes in the nuptial pads of *N. pleuraden*.
