# Supplementary material for: Love Hug—Functional Validation of Nuptial Pad-Secreted Pheromone in Anurans
Source: Animals (Basel). 2024 May 24;14(11):1550. doi: 10.3390/ani14111550 (PMC11171324; doi:10.3390/ani14111550)
Supplement: Supplementary file 1 [file animals-14-01550-s001.zip › Supplementary Materials.pdf]

Supplementary Materials

Love hug -- Functional Validation of Nuptial pad-secreted Pheromone in Anurans

Puyang Zheng[1,2,3#], Yuzhou Gong[4,5#], Bin Wang[1,3], Haoqi Yu[1,2,3], Sining Huang[1,3], Xun Liao[1,3], Jianping Jiang[1,3], Jianghong Ran[2], & Feng Xie[1, 3*]

1 Chengdu Institute of Biology, Chinese Academy of Sciences, Chengdu, Sichuan, 610041, China

2 Key Laboratory of Bio-Resources and Eco-Environment of Ministry of Education, College of Life Sciences, Sichuan University, Chengdu, Sichuan, 610065, China

3 University of Chinese Academy of Sciences, Beijing, 100049, China

4 Shanghai Natural History Museum, Branch of Shanghai Science & Technology Museum, Shanghai, 200041, China

5 School of Life Science, East China Normal University, Shanghai, 200062, China

# These authors contributed equally to the study.

*Corresponding author, E-mail: xiefeng@cib.ac.cn

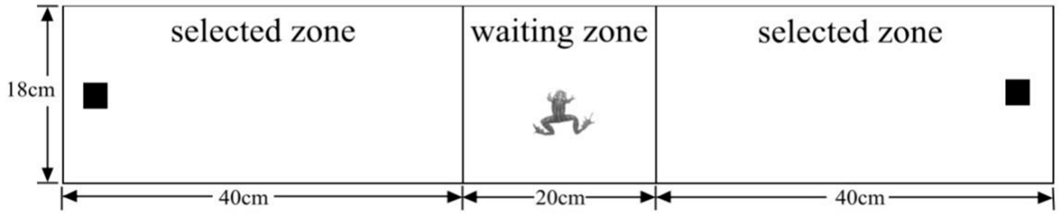

Supplementary Figure S1 Diagram of the setup of animal preference trials

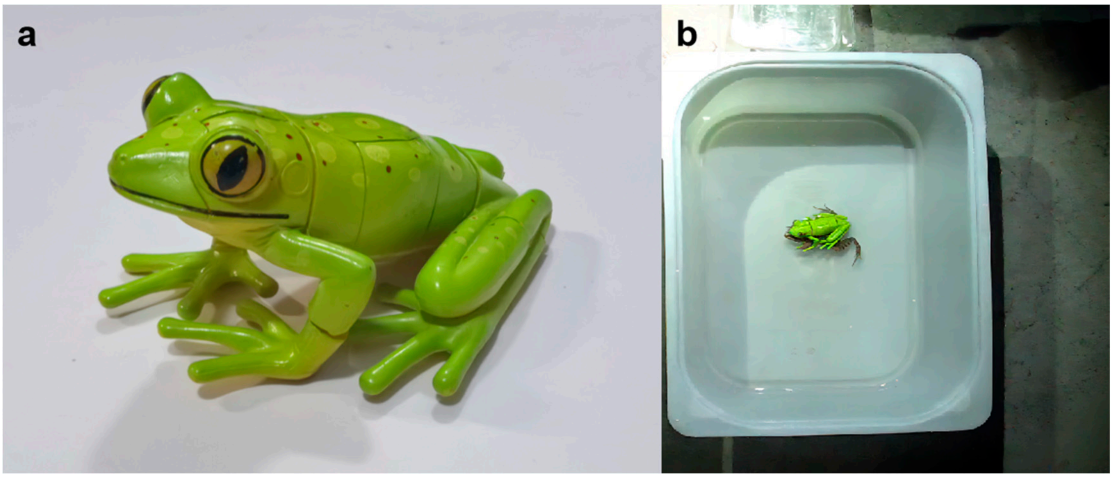

Supplementary Figure S2 (a) A model frog for amplexus experiments; (b) An experiment in progress

Note: The snout-vent length (SVL) of the model frog used in the experiment was 64.25mm and the body weight was 25.89g, which are similar to the adult male *N. pleuraden.*

Supplementary Table S1 The databases and softwares used for non-redundant transcripts function annotation

Database Name	Annotation software	E-value
NR (NCBI non-redundant protein sequences)	diamond v0.8.22	1e-5
NT (NCBI non-redundant nucleotide sequences)	NCBI blast 2.2.28+	1e-5
Pfam (Protein family, protein domain)	hmmscan 3.0	0.01
KOG/COG (Eukaryotic Ortholog Groups and Clusters of Orthologous Groups of proteins)	diamond v0.8.22	1e-3
Swiss-Prot (A manually annotated and reviewed protein sequence database)	diamond v0.8.22	1e-5
GO (Gene Ontology)	Blast2GO b2g4pipe_v2.5 (Götz et al., 2008)	1e-6
KO (KEGG Orthology database)	KEGG Automatic Annotation Server (KAAS) r140224	1e-10

Supplementary Table S2 Up-regulated genes in the nuptial pads of *N. pleuraden* and their annotation informations

(Excel table)

Supplementary Table S3 Go enrichment of Up-regulated genes in the nuptial pads of *N. pleuraden*

(Excel table)
